# Physical exercise recommendations for patients with chronic myeloid leukemia based on individual preferences identified in a large international patient survey study of the East German Study Group for Hematology and Oncology (OSHO #97)

**DOI:** 10.3389/fonc.2024.1345050

**Published:** 2024-02-21

**Authors:** Lina Hollenbach, Julia Rogahn, Philipp le Coutre, Susann Schulze, Lars-Olof Muegge, Jan Geissler, Julia Gruen, Christian Junghanss, Sabine Felser

**Affiliations:** ^1^ Department of Internal Medicine, Clinic III – Hematology, Oncology and Palliative Care, Rostock University Medical Center, Rostock, Germany; ^2^ Department of Hematology, Oncology, and Cancer Immunology, Campus Virchow‐Klinikum, Charité ‐ Universitätsmedizin Berlin, Berlin, Germany; ^3^ Krukenberg Cancer Center Halle, University Hospital Halle, Halle (Saale), Germany; ^4^ Department of Medicine, Medical Clinic II, Carl-von-Basedow-Klinikum, Merseburg, Germany; ^5^ Department of Internal Medicine III, Heinrich Braun Klinikum Zwickau, Zwickau, Germany; ^6^ LeukaNET/Leukemia-Online e. V., Riemering, Germany

**Keywords:** chronic myeloid leukemia (CML), physical exercise recommendation, health-related quality of life (QOL), myeloproliferative neoplasm (MPN), physical activity

## Abstract

**Background:**

Tyrosine kinase inhibitors (TKIs) have significantly lowered mortality of chronic myeloid leukemia (CML) patients adjusting life expectancy to that of the standard population. However, CML and its treatment with TKIs causes a high disease burden. Physical exercise (PE) could be a non-pharmacological approach to reducing these and improving quality of life.

**Purpose:**

The aim of this study was to determine the individual disease burden as well as PE preferences of CML patients and to deduce thereof specific PE recommendations.

**Methods:**

This multicenter survey was conducted in cooperation with the LeukaNET/Leukemia-patient network including CML patients aged ≥18 years (German Registry of Clinical Trials, DRKS00023698). The severity of selected symptoms was assessed using the adapted Myeloproliferative Neoplasms Symptom Assessment Form: 0 (absent), 1–30 (mild), 31–70 (moderate), or 71–100 (severe). Information about patients’ PE needs and preferences depending on their motivation was recorded.

**Results:**

A total of 212 questionnaires were analyzed (52% female, median age 54 years). The prevalence of moderate-to-severe symptoms was 49% for fatigue, 40% for musculoskeletal pain, and 37% for concentration problems. Other commonly reported symptoms included skin reactions (42%) and weight gain (24%). The proportion of overweight/obese patients was 52%. Half of all respondents requested more information regarding PE. Patients with CML preferred individual training (82%), located outdoors (71%), at home (47%), or in an indoor swimming pool (31%). Regarding the training frequency, sports-inactive patients preferred a frequency of 1–2 training sessions per week, whereas sports-active patients preferred 3–4 sessions per week (p <0.001). Sports-inactive patients preferred a training time of 15–45 minutes, while sports-active patients preferred 30–60 minutes (p = 0.002). Subsequently, PE recommendations were developed for patients with CML. Combined resistance and endurance training (moderate intensity twice per week for 30 minutes) was recommended for beginners. Obese patients should prioritize joint-relieving sports. To reduce the risk of skin reactions, direct sunlight and possibly water sports should be avoided, and UV protection should be used.

**Conclusion:**

Counseling and motivation of CML patients to be physically active should be part of the standard of care as well as support for implementation.

## 1 Introduction

Chronic myeloid leukemia (CML) belongs to the group of myeloproliferative neoplasms (MPN) (1). The incidence of CML is 1–2 cases per 100,000 adults, and men are affected slightly more often than women (2). The median age of patients with CML in western countries is about 57 years (3, 4). CML is characterized by an acquired chromosomal translocation, which leads to a constitutively active tyrosine kinase (BCR::ABL1) with unrestrained cell production, due to the absence of its regulatory mechanism. This particularly affects granulocytes in CML (5). Since BCR::ABL1 has been identified as the molecular defect responsible for the pathogenesis of CML, tyrosine kinase inhibitors (TKI) have been developed to suppress the activity of BCR::ABL1 (6). Without therapeutic intervention, CML progresses into a so-called blast crisis and, within 3–5 years, is almost always fatal (7). While treatment with hydroxyurea, interferon alpha, and stem cell transplantation were among the most commonly used therapies until 2000, tyrosine kinase inhibitor (TKI) therapy is now the standard of care (8). Thanks to treatment with TKI, CML can be permanently controlled, and patients with CML have a life expectancy similar to that of the general population (9–11). Consequently, the prevalence of patients with CML is continuously increasing (12). Along with the necessary long-term or potential lifelong use of TKI, many patients with CML suffer from a variety of disease- and therapy-related side effects accompanied by a sometimes high symptom burden, which impairs health-related quality of life (QoL) (13–17). The degree of expression of the different side effects and symptoms correlates with the different time periods of the disease and also depends on the generation of TKIs including their toxicity profiles (15, 18). The period of diagnosis is characterized by mild or rather nonspecific symptoms such as fatigue, a depressive mood, upper abdominal pain (due to potential splenomegaly), and B symptoms, which include weight loss due to increased metabolism (7, 8). In the first treatment period, which takes place during the initial weeks, a targeted therapy approach may lead to hematological adverse events associated with an increased risk of infection, bleeding, or anemia (8, 19). Anemia can lead to increased fatigue, headache, dizziness, and shortness of breath (18). The blood count is expected to return to normal and the spleen to a normal size after approximately three months (complete hematologic response) (20, 21). Furthermore, diarrhea and liver enzyme elevations may occur more frequently (18). A decrease in the metabolic rate with a simultaneous increase in appetite and increased water retention can lead to the onset of weight gain. The latter may also continue during the period of long-term TKI therapy (8) and lead to an increased body mass index (BMI). The occurrence of skin reactions is one of the most common side effects, which also occurs more frequently in the first months of TKI use and can continue during long-term TKI therapy. Furthermore, fatigue, concentration problems, memory impairment, bone and muscle pain, muscle cramps, and depression and anxiety are among the most common symptoms and side effects (14, 15, 22). Another health risk of long-term TKI therapy is the occurrence of cardiovascular events (8, 22, 23). Due to their typically young age, many CML patients on TKI therapy are still in the middle of life and face the challenge of coping with the demands of everyday life, such as maintaining a job. Consequently, treatment is currently more focused on alleviating the disease burden and improving QoL.

There is sufficient evidence that physical exercise (PE) can reduce symptoms such as fatigue, anxiety, and depression in cancer patients and improve perceived physical performance and QoL (24). While specific PE recommendations exist for patients with solid tumors, acute leukemia, and lymphoma as well as cancer survivors (24, 25), they are lacking for patients with CML and other MPNs (26, 27). Therefore, we analyzed the physical activity behavior of this patient cohort in a large multicenter study conducted within the East German Study Group for Hematology and Oncology (OSHO) (28). The results showed, among other things, that 65% of CML patients change their physical activity behavior because of the disease and the associated disease burden. While a small proportion said they were more conscious of or increased their physical activity in everyday life or sports, a significant proportion answered that they had reduced their physical activity. The majority of surveyed patients stated that they would like more information on this topic. Therefore, we performed more detailed analyses within the CML cohort and presented data beyond those published (28).

We conducted the current study to gain insight into (i) the disease burden, (ii) the level of information and request for more information about PE options of CML patients. In addition, we assessed (iii) PE preferences depending on demographic aspects and motivation for regular exercise. Subsequently, we (iv) developed symptom-based PE recommendations for CML patients considering the preferences.

## 2 Materials and methods

### 2.1 Study design, participants, and inclusion criteria

The design of the study has been published in detail earlier, see Felser et al. (28). Briefly, the study was designed as a multicenter, cross-sectional survey. It was approved by the Ethics Committee of the University of Rostock (A2020-0274) and registered with the German Registry of Clinical Trials (DRKS00023698). Patients ≥18 years of age with MPN (1) were eligible to participate in the survey. Patients with MPN from institutions of the OSHO (participating institutions can be found online at: https://www.frontiersin.org/articles/10.3389/fonc.2022.1056786/full#supplementary-material) were asked to participate and complete a hardcopy questionnaire (enrollment from January 2021 to September 2021). From April 2021 to September 2021, the study was amended by an online version of the survey, consisting of the same set of questions. The participants included patients of the LeukaNET/Leukemia-Online patient network as well as the German, Austrian, and Swiss MPN patient network. The data presented here include only patients with CML.

### 2.2 Questionnaire

#### 2.2.1 Demographic data

The self-administered survey comprised questions about gender, age, weight and height, education, family, and professional status. The BMI was calculated (body weight [kg]/height [m2]) and classified as <18.5, 18.5–24.9, 25.0–29.9, 30.0–34.9, 35.0–39.9, and ≥40.0, representing underweight, normal weight, overweight, and obesity grade I to III, respectively (29). Years of education (schooling) were categorized as ≤10 or >10 years.

#### 2.2.2 Clinical data

Age at diagnosis and the current therapies were assessed.

#### 2.2.3 Disease burden

Symptoms such as skin reactions or weight gain were surveyed. For some items, such as fatigue and musculoskeletal pain, an adapted version of the Myeloproliferative Neoplasm Symptom Assessment Form (MPN-SAF) (30), supplemented by other typical symptoms of CML, e.g., headache or diarrhea, was used. Each item was rated from 0 (absent) to 100 (worst imaginable). The symptom severity was divided into four categories: absent (0), mild (1–30), moderate (31–70), and severe (> 70).

#### 2.2.4 Information level

The patients’ level of information regarding the importance of and opportunities for physical activity and their need for information about this topic were recorded.

#### 2.2.5 Motivation to participate regularly in sports

The five stages of the transtheoretical model of behavioral change were used to determine the motivation to participate regularly in sports (31, 32). The questionnaire is provided online at: https://www.frontiersin.org/articles/10.3389/fonc.2022.1056786/full#supplementary-material). The answers were dichotomized: not regularly active in sports (stages 1, 2, and 3: precontemplation, contemplation, and preparation, respectively) or regularly active in sports (stages 4 and 5: action and maintenance, respectively).

#### 2.2.6 Physical exercise preferences

The patients were asked to indicate their PE preferences: the kind of training, location, frequency, and duration of each session.

### 2.3 Derivation of the exercise recommendations

To identify symptom-based exercise recommendations for CML patients, the method of integrative decision making was chosen (33). In the first step, sports scientists and physiotherapists deduced PE recommendations for patients with CML based on published data (PubMed search). Due to a lack of studies on the effects of exercise interventions in patients with MPN, primary evidence was deduced from studies of patients with other hematologic neoplasms or solid tumors. If no evidence-based symptom recommendations for cancer were available, the search was extended to other relevant patient cohorts. The deduced recommendations were supplemented with advice on common symptom management for CML. To assist CML patients in starting sports activities, we identified training possibilities/options considering the patients’ preferences. Subsequently, the PE recommendations compiled in step one were presented to oncologists, as well as patients from the LeukaNET/Leukemia-Online patient network. Oncologists and CML patients’ networks obtained the opportunity to express their subjective opinions or objections to the proposed PE recommendations. The focus was set on the avoidance of adverse events during or due to training. In the third step, all relevant objections were included in the initial PE recommendations. Acquiring the consent of all parties involved in the decision process, it was assumed that the new training recommendations were “safe enough to try”.

### 2.4 Statistical analysis

Continuous data are reported as the mean ± standard deviation, and categorical variables are presented as numbers and percentages. The Spearman correlation was used to determine the strength of linear correlations (Interpretation correlation coefficient r: |0.10| to |0.30| = weak, ≥|0.30| to |0.50| = medium, ≥|0.50| = strong). Mean differences were tested using the χ^2^ test (Fisher’s exact test). All data were analyzed using SPSS (version 25.0, IBM, Armonk, NY, USA). Statistical significance was assumed for *p*-values < 0.05.

## 3 Results

### 3.1 Demographic and clinical data

In total, we received 766 questionnaires. Due to missing information regarding diagnosis or too many missing details, we excluded 8% (n = 60) of the questionnaires. Thus, we included 706 questionnaires in the analysis. The CML cohort comprised 212 questionnaires, 62% (n = 132) in the hardcopy format and 38% (n = 80) in the online format. The patients’ demographic and clinical data are presented in **Table 1**. The analyzed cohort included 52% (n = 110) women. The median age of patients with CML was 54 years with a range of 18–87 years. According to the BMI calculation, 52% (n = 109) of the participants were overweight or obese. The proportion of overweight was higher in patients with a lower educational level, compared to patients with a higher educational level (63% vs. 43%, p = 0.009). At the time of the survey, 63% (n = 132) of patients with CML were working, of whom 8% (n = 10) were on sick leave. The first diagnosis of CML was a median of 5 years ago at the time of the survey (diagnosed between 1994 and 2021). At the time of the survey, 83% (n = 165) of patients self-reported taking TKIs, and 11% (n = 22) were in surveillance. Stem cell transplantation was given to 3% (n = 6) of patients in this cohort.

**Table 1:** Demographics, clinical data, and disease burden (n = 212).

Characteristic	n	Category	Values
**Gender**	210	Female Male	110 (52) 100 (48)
**Age [years]**	211		54 (18-87)
**BMI [kg/m^2^]**	209	< 18.5 18.5 – 24.9 25.0 – 29.9 30.0 – 34.9 35.0 – 39.9 ≥40	4 (2) 96 (46) 65 (31) 27 (13) 11 (5) 6 (3)
**School education [years]**	198	≤ 10 > 10	82 (41) 116 (59)
**Family status**	212	Single Married/living with a partner Other	51 (24) 150 (71) 11 (5)
**Professional status**	208	Working Retired Other	132 (63) 58 (28) 18 (9)
**Time since diagnosis [years]**	175		5 (0-27)
**Current therapy**	200	Tyrosine kinase inhibitors Surveillance only Cytostatics Interferon alpha	165 (83) 22 (11) 7 (3) 6 (3)
**Disease burden**	208	Skin reactions Weight gain Splenomegaly Unintentional weight loss Bleeding tendency	88 (42) 49 (24) 20 (10) 17 (8) 14 (7)

Data are presented as the number of participants (%) for categorical variables and as median (range) for continuous variables.

n, number of patients; BMI, body mass index.

### 3.2 Disease burden

**Figure 1:**
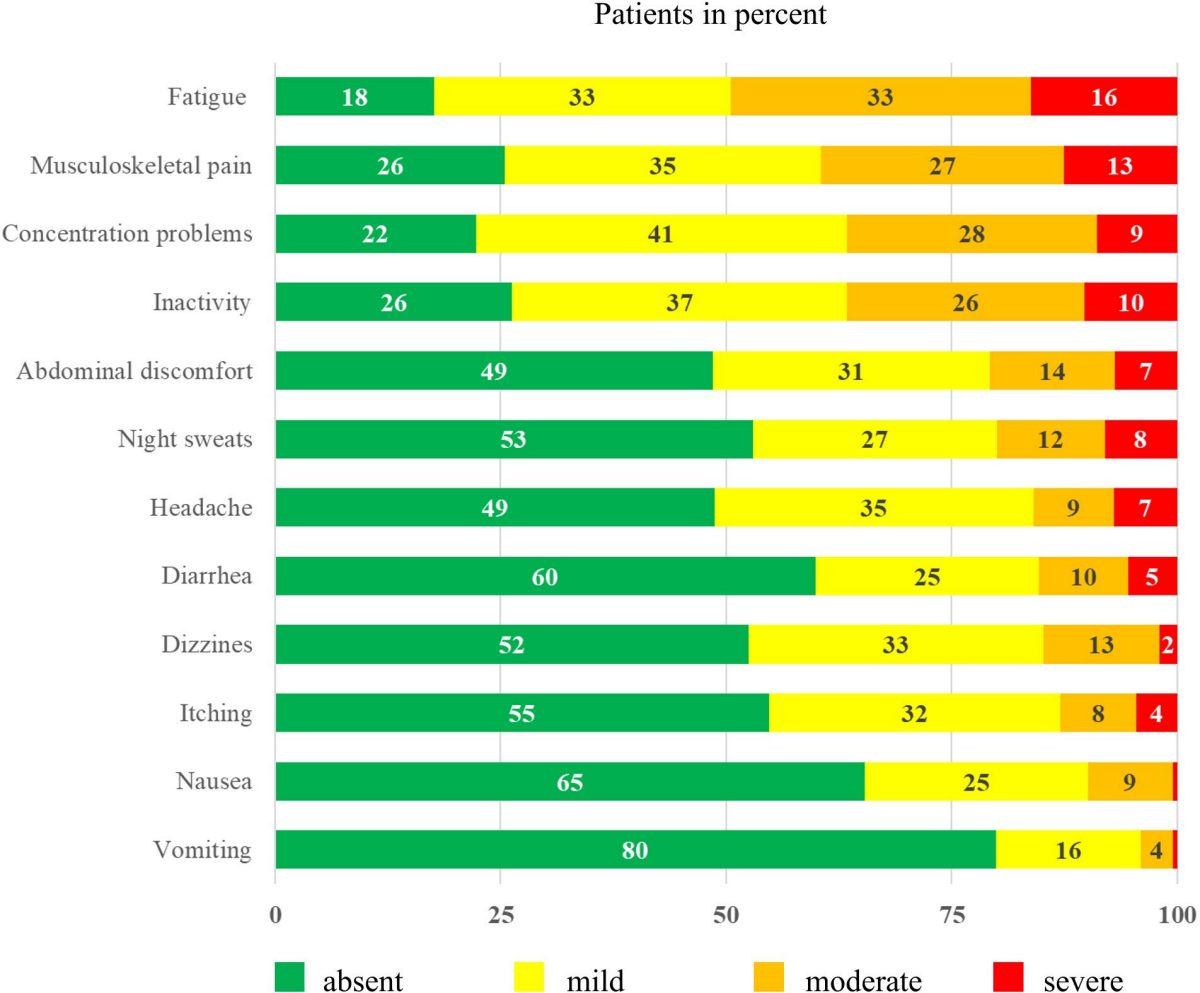
Symptom severity of patients with chronic myeloid leukemia* (n = 212) *Surveyed using an adapted version of the Myeloproliferative Neoplasm Symptom Assessment Form (MPN SAF) (34). Each item was scored on a scale from 0 (absent) to 100 (worst imaginable).

The frequencies of typical symptoms are shown in **Table 1**. The most frequently mentioned symptoms were skin reactions (42%, n = 88) and weight gain in the last three months (24%, n = 49). **Figure 1** provides an overview of the severity of selected symptoms. The most common symptoms of moderate-to-severe severity included fatigue (49%), musculoskeletal pain (40%), and concentration problems (37%). All three symptoms showed medium to strong correlations (*p <*0.001) and correlated with inactivity (*p <*0.001), with fatigue showing the strongest correlation (r = 0.749).

### 3.3 Information level

Nearly one-third (32%) of CML patients felt inadequately informed about the importance of and opportunities for physical activity in relation to their disease. Of these patients, 92% were interested in more information on this topic. Similarly, 30% of patients informed on the topic said they would like to receive more information. Consequently, 50% of all CML patients interviewed requested more information on physical activity. Differences related to demographic parameters did not appear.

### 3.4 Motivation to participate regularly in sports

The question about motivation to participate regularly in sports was answered by 89% (n = 188) of the patients with CML. The analysis of the stages of behavioral change revealed that 31% (n = 58) of them were not action oriented (precontemplation stage), and 26% (n = 49) were in the contemplation or preparation stage. In total, 43% (n = 81) of patients with CML reported regular participation in sports (action and maintenance stages). There were no differences in the information level or demographic parameters between the sport-active and the sport-inactive patients.

### 3.5 Physical exercise preferences

**Table 2** presents an overview of the PE preferences of patients with CML depending on motivation to participate in regular sports.

**Table 2:** Physical exercise preferences depending on motivation for regular sports.

Questions	Total cohort n = 199 [n (%)]	Not active in sports n = 107 [%]	Active in sports n = 81 [%]	*p*-value χ^2^-test
Which kind of training would you prefer?^†^				
**Individual training** **Group training**	155 (82) 66 (35)	77 37	90 32	0.029* 0.527
Which location would you prefer?^†^				
**Outdoor** **At home** **Indoor swimming pool** **Gym** **Physiotherapy** **Sports hall** **Other**	140 (71) 93 (47) 61 (31) 59 (30) 35 (18) 20 (10) 16 (9)	71 45 35 26 20 13 -	76 55 30 39 13 8 -	0.499 0.230 0.525 0.077 0.228 0.329
What is your preferred training frequency?				
**[times per week]**				<0.001**
**1 – 2** **3 – 4** **> 4**	110 (57) 62 (32) 20 (10)	69 26 5	39 42 19	
Which time per session would you prefer?				
**[minutes]**				0.002*
**< 15** **15 – 30** **30 – 45** **45 – 60** **> 60**	21 (11) 50 (26) 49 (25) 51 (26) 22 (11)	16 30 25 19 9	2 19 28 35 16	

n; number of patients.

^†^ multiple response possible; bold: statistically significance, *p <0.05, **p <0.001.

#### 3.5.1 Kind of training

Overall, this patient cohort prefers individual training. Moreover, a significantly higher proportion of those who are sport-active, compared to those who are sport-inactive reported a preference for individual training (90% vs. 77%, *p* = 0.029). The preference for group training was comparable in both groups and involved a total of 35% of the respondents. Women were more likely than men to indicate a preference for group training (42% vs. 27%, *p* = 0.046). Education-dependent differences with regard to the kind of training were also evident. While 90% of patients with a higher level of education indicated a preference for individual training, the proportion of patients with a lower level of education was 69% (*p <*0.001). The latter group tended to prefer group training more often (43% vs. 30%, *p* = 0.081).

#### 3.5.2 Training location

Regardless of the level of motivation to exercise regularly, training outdoors (71%), at home (47%), in an indoor swimming pool (31%), or in a gym (30%) is preferred. Differences were found depending on age and educational level. Thus, younger patients with CML (<60 years) preferred outdoor training more often than older ones (≥60 years) (76% vs. 60%, p = 0.033) and tended to train in a gym more often (34% vs. 19%, *p* = 0.052). Older patients, on the other hand, more often preferred physiotherapy as a training location (29 vs. 14%, *p* = 0.021). While 81% of patients with higher education levels reported preferring outdoor training, this proportion was 58% for patients with lower education levels (*p <*0.001). In patients with a lower educational level, physiotherapy was a preferred training location in contrast to patients with higher educational level (9% vs. 30%, *p <*0.001).

#### 3.5.3 Frequency and time per session

With regard to the amount of exercise, there were significant differences between sports-active and sports-inactive patients. Of the sports-inactive patients, 69% reported preferring a frequency of 1–2 times/week, while 31% preferred ≥3 times/week. By contrast, 39% of the sports-active patients preferred a frequency of 1–2 times/week and 61% preferred a frequency ≥3 times/week (*p <*0.001). Regarding the session duration, the sports-inactive patients preferred 15–45 minutes, while the sports-active patients preferred 30–60 minutes (*p* = 0.002). Although the proportion of sports-active patients was independent of education level (43% vs. 45%, *p* = 0.760), group differences emerged in terms of the preferred training volume. For example, the proportion of those who preferred training 1–2 times/week was higher in patients with lower education levels than in patients with higher education levels (68% vs. 49%, *p* = 0.049). In addition, patients with higher levels of education preferred longer session durations than patients with lower levels of education (*p* = 0.016).

### 3.6 Exercise recommendations for patients with chronic myeloid leukemia

PE with CML patients should focus on the symptoms of fatigue and musculoskeletal pain. Fatigue significantly limits QoL in CML patients (35) and is associated with muscle discomfort, which in turn contributes to decreased disease control (34). In addition, fatigue is often associated with memory and concentration problems (36). Recent data suggest that fatigue and concentration problems are independent predictors of falls in MPN patients (37). PE recommendations should likewise consider weight gain/obesity and skin reactions. Unwanted weight gain, as well as skin reactions can alter the appearance and body image of CML patients (38). Thus, obesity not only increases the risk for metabolic disorders (39) but also increases the likelihood of a wide range of psychiatric disorders, including anxiety and depression (40). Visible skin symptoms can also have psychosocial consequences and affect QoL and self-esteem (41–44). Below, we provide PE recommendations for patients with CML in relation to the previously mentioned symptoms. To help sport-inactive CML patients get started in sport activities, we highlight training options that take into account the preferences of these patients. **Table 3** summarizes the PE recommendations.

**Table 3:** Exercise recommendations for patients with chronic myeloid leukemia.

Category of the FITT principle^†^	General recommendations adapted to patients´ preferences
Frequency	At least 2 sessions per week
Intensity	Moderate, progressive
Time	At least 30 minutes per session, progressive
Type	Combined resistance-endurance training or separate from each other
Symptoms/side effects	Special recommendations
Fatigue/concentration problems	Yoga, tai-chi/qigong as an alternative or supplement to combined resistance-endurance training
Anxiety/depression	Emphasis on endurance training or yoga, tai-chi/qigong
Musculoskeletal pain	Emphasis on resistance training
Weight gain/obesity	Joint-relieving sports (e. g. swimming, walking, cycling), muscle hypertrophy
Edema	Aqua-therapy or exercising in water
Skin reactions	Avoiding direct sunlight (possibly also water), protect skin with clothing, using UV-protection
Increased risk of infection	Individual training, hand disinfection and face mask during group training; waiving indoor swimming pools
Splenomegaly	Equipment training, avoiding contact/ball sports
Bleeding tendency	Equipment training, avoiding contact/ball sports
Dizziness	No quick changes of position, training in company
**Notice:** Higher age, overweight, fatigue or concentration problems are independent **risk factors for falls** (37). Recommendations: (in addition) coordination training incl. balance training and the use of fall prevention strategies (e.g., poles when walking).	
Preferred kind of training	Special recommendations (examples)
Individual training	Indoor: circuit training with own body weight or small equipment, ergometer training, swimming, aqua jogging Outdoor: circuit training with own body weight and included parts of rapid walking, running, cycling or skipping rope; nordic walking, skiing, rowing
Group training	Indoor: various rehabilitation or fitness courses also aqua jogging/gymnastics, dancing, yoga Outdoor: walking, running, cycling or circuit training in company of others

^†^FITT is an acronym for frequency, intensity, time and type. Adjustments required for (multi)comorbidity; generally medical approval should be obtained before starting exercise.

#### 3.6.1 Fatigue and psychological symptoms

There is strong evidence that cancer patients can alleviate symptoms such as fatigue, anxiety, and depression with two to three sessions of moderate-intensity endurance training or combined resistance-endurance training per week (24, 45). Fatigue appears to be more responsive to moderate- to vigorous-intensity exercise, and it is unlikely that mild-intensity PE can help alleviate fatigue (24). Meta-analyses in adults with hematologic diseases showed moderate effects of the aforementioned exercise types with respect to fatigue and small effects with respect to depression (45). Especially in the case of psychological symptoms, the focus should be on endurance training (24). Exercise forms such as yoga, tai chi, and qigong also appear to be suitable for alleviating the symptom burden and improving QoL in cancer patients (46, 47). Due to the small number of studies with patients with MPN or other hematologic malignancies, concrete conclusions about effectiveness are not possible. However, in feasibility studies with Philadelphia chromosome-negative MPN patients, psychological and physical symptoms were alleviated by 50 minutes of yoga per week (48, 49).

#### 3.6.2 Musculoskeletal pain

Regarding the effects of PE on treatment-related pain in cancer patients, the available studies suggest that resistance training or combined resistance-endurance training may contribute to pain relief (50, 51). However, a recent review and meta-analysis found the overall risk of bias for most studies was rated as some concern and the grading of evidence certainty was low (52). It is possible that endurance and/or resistance training with blood flow restriction could also have beneficial effects in patients with CML suffering from chronic pain, as has been shown in other patient cohorts (53, 54).

#### 3.6.3 Weight gain/obesity

Many CML patients often gain weight due to the side effects of TKIs (55) or are already overweight before the onset of disease (56). Therefore, we suggest muscle hypertrophy training to regulate body weight. Increasing lean mass increases energy metabolism (57). Additional endurance training or the combination of both may have beneficial effects on the immune system in patients with hematologic malignancies (58). Because obesity is also associated with osteoarthritis (59) and can cause or exacerbate joint pain (60), affected individuals should choose joint-friendly sports such as cycling, swimming, or water aerobics. Both weight reduction and the positive immunomodulatory effects of moderate exercise (61, 62) may have analgesic effects. For edema, training in water is ideal, as the water pressure supports lymphatic drainage (63).

#### 3.6.4 Recommendations considering the preferences

In general, at least two 30-minute sessions of combined resistance and endurance training per week are recommended for CML patients starting exercise. Alternatively, strength and endurance training can be performed separately. Circuit training of moderate intensity is recommended, with progressive load adjustments. With appropriate exercise selection, consisting of 6–12 exercises for different muscle groups, and the inclusion of endurance exercises, such as fast walking, running, or cycling, circuit training is time-saving and comprehensive. The optimal time for inexperienced CML patients to start exercising appears to be the transition to the period of long-term TKI therapy. Sports-experienced CML patients should continue their training during the first treatment period and adapt the training volume and intensity to the current conditions. During the period of long-term TKI therapy, the amount of exercise (frequency and/or time) should be slowly increased. A minimum of three training sessions or 150 minutes per week should be aimed for. Combined resistance-endurance training can be performed independently outdoors or at home using the patient’s own body weight and/or small equipment, such as elastic bands or dumbbells. Outdoors, stairs, benches, or railings can be used for exercise. CML patients who prefer indoor swimming pool training can combine aqua jogging or swimming with strength exercises in the water. Only CML patients with an increased risk of infection should avoid indoor swimming pools. If skin reactions occur, depending on the trigger, activities in (chlorinated) water or when exercising outdoors, direct sunlight should be avoided. The skin should then be protected by clothing (64). In general, appropriate sun protection should be used during outdoor activities to prevent melanoma (65).

CML patients who prefer group training can, in principle, participate in all sports. Only patients with splenomegaly or a bleeding tendency should avoid ball and contact sports or sports with a high risk of falling or injury. Since there is no evidence to date on whether training with free weights is safe for patients with splenomegaly, low-injury training on equipment (weight-training machines and ergometers) is recommended for them. Otherwise, all rehabilitation and fitness courses offered in physiotherapies, fitness studios, or sports clubs are suitable. Patients at risk of infection who participate in group exercise should avoid direct contact with others, disinfect their hands, and wear a face mask. Because preliminary data suggest that fatigue and concentration problems may be independent risk factors for falls, along with older age and higher BMI (37), CML patients who combine one or more predictors should consider fall prevention strategies, e.g., poles when walking. Integrating balance exercises into circuit training or performing short, independent training sessions with coordination exercises is also possible. We especially recommend that CML patients (I) with no previous experience in sports, (II) with other concomitant diseases, and/or (III) with an increased cardiovascular risk (66), obtain medical clearance before starting PE and begin supervised sports programs. For some outcomes, particularly psychological symptoms, supervised interventions are more effective than unsupervised home exercise programs (24, 67). CML patients, like other patient cohorts, have the option of PE by prescription, e.g., for physiotherapy, physiotherapy on machines, or rehabilitation sports.

#### 3.6.5 Further advice

Patients who experience dizziness should avoid rapid changes of position during exercise and ideally should not exercise alone. Symptomatic relief from muscle cramps can be achieved with quinine-containing beverages (e. g. tonic water or bitter lemon) (16).

## 4 Discussion

Here, we present the first study to investigate the level of information and information needs about physical activity and PE preferences among patients with CML. Further, novel, specific PE recommendations for patients with CML were developed, which are primarily aimed at CML patients in the chronic phase and undergoing first-line therapy. A key finding is that patients with CML, comparable to patients with polycythemia vera (68), have a high need for information on physical activity, regardless of the level of motivation to exercise regularly. The frequently young age at diagnosis in combination with employment, increases the relevance of this topic. Therefore, specific/individual PE recommendations should be integrated into the treatment of patients with CML. An optimal time for consultation could be as early as possible after diagnosis, as recommended by the American Cancer Society in its guideline for all cancer patients (69). From our point of view, another good time would be the transition to long-term TKI therapy, which is particularly suitable for CML patients who are inexperienced or sports-inactive. In this context, treating physicians should educate patients with CML about the real risk of infection and ways to reduce the risk of infection during PE, as the fear of such events is a barrier to physical activity (28). Clarification of German-language patient guides (70, 71), which contain little CML-specific information on PE, in contrast to English-language guides (38), could contribute to knowledge transfer regarding the topic. As indicated by surveys of other patient cohorts alternative effective methods for PE counseling and instruction could be face-to-face or technology-based (e.g. internet, email) information exchange with a PE specialist from cancer centers (72–75).

Another key finding of this study is that the PE preferences of CML patients, especially those who are inactive, are significantly below the general recommendations of international professional societies, which is consistent with the findings of Vallerand et al. (76). For example, the American College of Sports Medicine and the World Cancer Research Fund/American Institute for Cancer Research, recommend at least three training sessions per week and at least 150 min of moderate-intensity physical activity (24, 77). However, these recommendations apply predominantly to cancer survivors after completion of cancer therapy. For patients with CML, exercise criteria, including both volume and intensity, should be adjusted to symptoms and side effects, specifically during the first treatment period. With the transition to long-term TKI therapy, CML patients should be motivated to adhere to PE recommendations. Since one in three patients with CML reported not being action-oriented with regard to regular exercise, this could be a challenge for the counseling/treatment team. Behaviors become entrenched over time and are difficult to change (78). To make matters worse, fatigue and pain are barriers to physical activity (79, 80). Primarily collected data on patients with solid tumors, show that the willingness to take up PE programs and the respective preferred training volumes are higher after completion of treatment than during treatment (74, 81, 82). This indicates that the therapy itself and the associated side effects are key barriers to physical activity/PE. An additional complication for CML patients is their likelihood to have comorbidities in a higher extend than the general population (83). Accordingly, these comorbidities can have a negative impact on exercise behavior. Further, there are several other barriers to PE, including older age, distance from structures, lack of motivation, lack of time, lack of information, as well as physical, personal and emotional problems (73, 82). Therefore, the involvement of a psychologist to implement and maintain changes in exercise behavior could be beneficial. There is also evidence that barriers decrease with increasing PE experience/habits (84). CML patients with low levels of education often indicated a preference for group exercise or even physical therapy. It is possible that these patients could be motivated to exercise regularly in a group setting through appropriate referrals/prescriptions for physical therapy or rehabilitation sports. In addition, all patients with CML, but especially those who cannot be motivated to engage in regular sports activities, should be encouraged to reduce sedentary activities and increase their physical activity in daily life. This could have positive effects on fatigue and QoL in CML patients, as shown by data on Patients with Philadelphia chromosome-negative MPNs (85). Reducing sedentary behavior, decreases the risk of diseases such as heart disease and type 2 diabetes (86, 87). In addition, high levels of physical activity after diagnosis reduce the risk of cancer-specific mortality, as shown in studies of solid tumors (65). Although this effect has not yet been demonstrated in hematologic diseases (45), we hypothesize that these benefits may also apply to CML patients. This is supported by the fact that CML patients have a higher prevalence of comorbidities compared to the general population, especially cardiovascular diseases and their risk factors such as hypertension, diabetes and obesity (83). These comorbidities not only increase the risk of side effects such as arterio-occlusive events (88), but are now also the leading cause of death in CML patients treated with TKIs (89). Since comorbidities influence the choice/side effects of TKIs, they have a significant impact on overall survival (89, 90). However, studies with large numbers of cases and multi-year study durations are needed to make meaningful conclusions about the effects of physical activity on mortality. Increasing daily physical activity could help overweight patients with CML to regulate their body weight, which in turn could have a positive impact on symptom burden (91). It is also possible that daily physical activity and weight reduction could reduce the risk of falls (28, 37). Nutritional counseling can support the plan to change diet and reduce weight. The extent to which weight normalization is possible with TKI use, especially with imatinib, needs to be investigated separately, since imatinib also affects fat metabolism, among other things (55). Because CML patients with low educational levels are more likely to be overweight/obese, special attention and support should be given to this cohort.

A comparison of the exercise preferences of CML patients with other cancer entities reveals similarities but also clear differences. In line with the fact that older age is a barrier to PE, 75% of adolescents and young adults with cancer prefer an exercise frequency of ≥3 times per week (82). In contrast, Fournier et al. (92) found that the proportion of cancer patients above 70 years undergoing therapy preferring 1 - 2 PE sessions per week is 77%, which is 20% higher compared to CML patients. The proportion of CML patients preferring an ≥ 30 minute exercise per session was 62% - comparable to the results of Fournier et al. (92). Blaney et al. (81) surveyed 456 cancer survivors, including 64% breast cancer patients, of whom only 30% stated that they preferred an exercise time of >30 min. In patients with brain tumors, the proportion of those who preferred an exercise time of >30 min ranged from 18% to 44%, depending on whether they were undergoing treatment or had already completed it (74). Preferences also differed with regard to the training location depending on the studied cohort. While 47% of CML patients stated that they preferred to exercise at home, the literature reports between 20% and 83% (72, 74, 82, 83, 89, 90). While 80% of patients with incurable cancer prefer to exercise alone and unsupervised (93), other patient cohorts, including head and neck cancer patients (84) and cancer survivors (72) prefer supervised exercise programs. The preference of CML patients to exercise individually and outdoors may indicate that “walking” is a popular form of exercise, as also described in other patient cohorts (81, 82, 94–96). However, the proportion here varies between 22% in patients with incurable cancer (93) and 83% in cancer survivors during the COVID-19 pandemic (94). Consequently, training preferences differ not only depending on the patient studied cohort, but also, as can be seen in the results of the CML patients, on demographic parameters such as age and gender (82, 92–94, 97), educational level (94, 98), clinical parameters such as under therapy vs. time after therapy (74, 95), performance status (93), current PA behavior/level (82, 94, 98) and psychological constructs associated with behavior, such as self-efficacy or perceived behavioral control (96). According to these findings, CML patients require specific exercise programs taking into account their individual interests and needs.

Because few studies are currently available on the effects of exercise interventions in patients with CML or MPN (49, 99, 100), we based our derivation of PE recommendations on evidence from studies in patients with other hematologic neoplasms or solid tumors. In addition, to our knowledge, no study results are available to date on whether exercise activities in patients with CML lead to increased skin reactions, e.g., sweating, friction against clothing, or chlorinated water. Therefore, the feasibility, safety, and efficacy of the new PE recommendations for patients with CML should be evaluated and, if appropriate, substantiated in subsequent prospective, longitudinal studies.

### 4.1 Strengths and limitations

Despite its low incidence, a large cohort of patients with CML in German-speaking regions could be recruited. Due to the survey design, there are unavoidable limitations that must be considered when interpreting the data. First, as the survey was voluntary and also conducted online, it is possible that mainly younger and sport-affine patients with CML participated. Consequently, the proportion of sport-inactive patients with CML might be higher than the data suggest. Second, we conducted the survey during the COVID-19 pandemic, so exercise preferences might be biased in terms of type and ambience (location) (94). Third, with respect to the overall cohort, patients with MPN, we used the MPN-SAF to assess symptom burden. This was supplemented with other symptoms of CML so that the main symptoms relevant to exercise therapy could be recorded and evaluated. CML-specific assessments should be used in subsequent studies. Fourth, detailed data about the respective TKIs or cytostatic drugs the CML patients were taking at the time of the survey was not recorded. Consequently, subgroup analyses related to symptom burden, motivation, and PE preferences was not focused. Since exercise recommendations are based on symptoms regardless of disease and therapeutic protocol, this missing information has in the current state no impact.

## 5 Conclusion and outlook

In conclusion, the current study has shown that information on physical activity is important for patients with CML. Physical activity counseling should become an integral part of the treatment plan, as patients with CML can benefit from physical activity in many ways. PE planning should be individualized according to the different patient preferences of, taking into account influencing demographic variables and existing PE experience. In addition, fears of potential barriers should be reduced helping to overcome these barriers. Specifically, in CML patients with lower educational levels, prescribing PE could contribute to behavior change. For the first time, we have outlined PE recommendations based on symptoms, to provide specific guidance for patients with CML. Prospective studies evaluating the feasibility, safety, and efficacy of the proposed PE recommendations are needed to ultimately provide evidence-based recommendations for patients with CML. In addition, it should be investigated whether providing knowledge about the opportunities and effects of physical activity leads to changes in PE behavior in patients with CML.

## Data availability statement

The raw data supporting the conclusions of this article will be made available by the authors, without undue reservation.

## Ethics statement

The studies involving humans were approved by Ethics Committee of the University of Rostock. The studies were conducted in accordance with the local legislation and institutional requirements. The participants provided their written informed consent to participate in this study.

## Author contributions

LH: Conceptualization, Data curation, Formal analysis, Writing – original draft, Writing – review & editing. JR: Data curation, Formal analysis, Writing – original draft, Writing – review & editing. Pl: Investigation, Writing – review & editing. SS: Investigation, Writing – review & editing. LM: Investigation, Writing – review & editing. JG: Investigation, Writing – review & editing. JuG: Data curation, Investigation, Writing – review & editing. CJ: Conceptualization, Formal analysis, Supervision, Writing – review & editing. SF: Conceptualization, Data curation, Formal analysis, Funding acquisition, Methodology, Project administration, Resources, Supervision, Writing – original draft, Writing – review & editing.
